# STAT3, a Hub Protein of Cellular Signaling Pathways, Is Triggered by β-Hexaclorocyclohexane

**DOI:** 10.3390/ijms19072108

**Published:** 2018-07-20

**Authors:** Elisabetta Rubini, Fabio Altieri, Silvia Chichiarelli, Flavia Giamogante, Stefania Carissimi, Giuliano Paglia, Alberto Macone, Margherita Eufemi

**Affiliations:** 1 Department of Biochemical Sciences, A. Rossi Fanelli, Sapienza University, P.le A. Moro 5, 00185 Rome, Italy; elisabetta.rubini@uniroma1.it (E.R.); fabio.altieri@uniroma1.it (F.A.); silvia.chichiarelli@uniroma1.it (S.C.); flavia.giamogante@uniroma1.it (F.G.); stefania.carissimi@uniroma1.it (S.C.); giuliano.pag@gmail.com (G.P.); alberto.macone@uniroma1.it (A.M.); 2 Istituto Pasteur-Fondazione Cenci Bolognetti, Sapienza University, P.le A. Moro 5, 00185 Rome, Italy

**Keywords:** STAT3, β-hexaclorocyclohexane (β-HCH), signal transduction, energy metabolism

## Abstract

Background: Organochlorine pesticides (OCPs) are widely distributed in the environment and their toxicity is mostly associated with the molecular mechanisms of endocrine disruption. Among OCPs, particular attention was focused on the effects of β-hexaclorocyclohexane (β-HCH), a widely common pollutant. A detailed epidemiological study carried out on exposed population in the “Valle del Sacco” found correlations between the incidence of a wide range of diseases and the occurrence of β-HCH contamination. Taking into account the pleiotropic role of the protein signal transducer and activator of transcription 3 (STAT3), its function as a hub protein in cellular signaling pathways triggered by β-HCH was investigated in different cell lines corresponding to tissues that are especially vulnerable to damage by environmental pollutants. Materials and Methods: Human prostate cancer (LNCaP), human breast cancer (MCF-7 and MDA-MB 468), and human hepatoma (HepG2) cell lines were treated with 10 μM β-HCH in the presence or absence of specific inhibitors for different receptors. All samples were subjected to analysis by immunoblotting and RT-qPCR. Results and Conclusions: The preliminary results allow us to hypothesize the involvement of STAT3, through both its canonical and non-canonical pathways, in response to β-HCH. Moreover, we ascertained the role of STAT3 as a master regulator of energy metabolism via the altered expression and localization of HIF-1α and PKM2, respectively, resulting in a Warburg-like effect.

## 1. Introduction

Organochlorine compounds are widely distributed in the environment and several studies link their basic molecular mechanism of endocrine disruption [1,2] with the onset of many pathological conditions such as chronic inflammatory processes, cardiovascular diseases, neurological and metabolic disorders, and oncogenesis [3,4,5]. The toxicity of organochlorine compounds is related to their physicochemical properties, because these pollutants belong to a group of organic compounds, known as “persistent organic pollutants” (POPs), that are resistant to degradation or biodegradation and that can be bioaccumulated into adipose tissue because of their lipotropic properties and great stability [6]. Observational epidemiological studies, carried out on population at high risk of exposure, revealed that POPs may play an important role in the development of a chronic inflammatory state by interfering with pathways associated with essential cellular processes and homeostasis [7]. Acute inflammation represents one of the early responses to injury but, if the causal insult becomes persistent, it may progress with a long chronic phase; although acute inflammation is necessary to rid the organism of foreign pathogens, chronic inflammation can be harmful, damaging to normal tissues, and may even develop into cancer [8].

It is well known that an altered activation of several signaling pathways triggered by cytokines, growth factors, and oxidative stress may sustain inflammation [9]; thus, it seems reasonable to investigate the involvement of the protein STAT3 (signal transducer and activator of transcription 3) in the mechanisms linking the effects of POPs to the pathological features typical of a chronic inflammatory state [10]. The pleiotropic role of STAT3 as a “molecular hub” in cellular signaling networks and in carcinogenesis is widely described in scientific literature [11,12]. In addition, recent studies provided evidence that STAT3 is a master regulator of energy metabolism, in particular under oxidative stress conditions [13,14]. STAT3 plays a key role in a vicious cycle related to the Warburg Effect [15], a metabolic reprogramming, resulting in an enhanced lactate production as a source of energy, even in normoxia, rather than the more efficient mitochondrial oxidative phosphorylation pathway. This effect occurs as an adaptation mechanism necessary to support the biosynthetic requirements of uncontrolled proliferation in cancer cells [16].

Considering the involvement of POPs in many different diseases [5], it is conceivable to hypothesize that they may exert the toxic effects not only by interfering with the activity of endogenous hormones, but also through the activation of “non-genomic” pathways [17,18], which cross-talk with other signaling cascades involving the protein STAT3. The aim of this study is to investigate the STAT3-mediated molecular mechanisms at the basis of POPs-induced toxicity. Attention was focused on the effects of the β-isomer of hexaclorocyclohexane (β-HCH), a synthesis byproduct of the insecticide lindane (γ-HCH), characterized by a high lipid solubility, environmental persistence, and long biological half-lives in human tissues [19,20]. Although β-HCH shows a worldwide distribution (Italy, Spain, Kazakhstan, Canada, India, China, Russia, Poland, Germany, Argentine) [21], its biological effects have not been largely studied.

The role of STAT3 as a modulator of intracellular processes such as apoptosis, proliferation, cellular growth, angiogenesis, and metabolic reprogramming has been investigated through the analysis of both its canonical and non-canonical pathways [22]. Hence, the evaluation of the impact of β-HCH on cellular conditions was carried out on a panel of cell lines representing different human tumor types associated with the expression and activation of specific receptors that are related to STAT3 activity.

## 2. Results

### 2.1. β-HCH Triggers Key Molecular Pathways in Different Cancer Cells

To address if STAT3 could act as a hub protein involved in the signaling pathways and cellular responses triggered by β-HCH and to verify if the adverse effects of β-HCH can be attributed to the activation of non-genomic pathways that cross-talk with other signaling cascades, time-course assays were first performed, exposing to β-HCH the following cell lines: human breast cancer (MDA-MB468) (EGFR+), human hepatoma (HepG2) (JAK2+), human prostate cancer (LNCaP) (AR+), and human breast cancer (MCF-7) (Her2+). The experimental concentration of β-HCH (10 μM) was extrapolated both from environmental–epidemiological studies carried out on the exposed population living throughout the “Valle del Sacco”, south of Rome [23,24], and from previous in vitro studies [25,26].

First of all, the effect of β-HCH on cell proliferation and viability was tested on all utilized cell lines. No appreciable reduction in cell viability was observed when exposing cells to 10 μM β-HCH, while β-HCH showed some proliferative effects (Figure S1 of supplementary data). These data are consistent with previous observations on MCF-7 cells [27]. Only at higher β-HCH concentrations a reduction of cell viability can be observed (Figure S2 of supplementary data).

**Figure 1:**
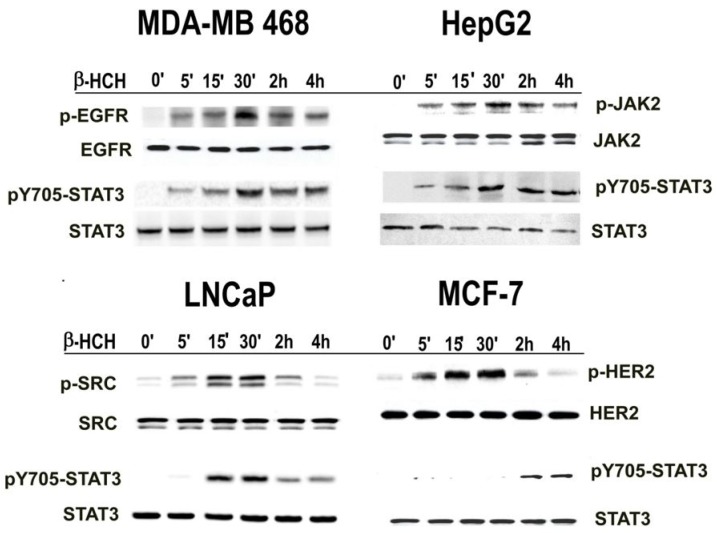
Evaluation of the signaling pathways triggered by β-hexaclorocyclohexane (β-HCH). Immunoblot analysis of the time-course assay performed on human prostate cancer (LNCaP), human breast cancer (MCF-7 and MDA-MB 468), and human hepatoma (HepG2) cells treated with 10 μM β-HCH. Samples were analyzed for signal transducer and activator of transcription 3 (STAT3) and each cell line for a specific membrane or membrane associated tyrosine kinase receptor: EGFR in MDA-MB 468 cells, JAK2 in HepG2 cells, SRC in LNCaP cells and HER2 in MCF-7 cells. Both unmodified and the corresponding phosphorylated form were detected for each protein using specific antibodies.

The activation of STAT3 upon β-HCH treatment was followed by immunoblotting analysis. The cell lines used in this study were selected on the basis of specific overexpressed receptors, potentially involved in STAT3 activation: membrane and membrane associated tyrosine kinase receptors (EGFR in MDA-MB 468, JAK2 in HepG2, and HER2 in MCF-7 cells) and cytoplasmic non-receptor tyrosine kinases (SRC in LNCaP cells). Total proteins were extracted from treated cells and then analyzed by Western blotting using specific antibodies against STAT3 and the different cellular receptors. Both unmodified and the corresponding phosphorylated (activated) forms were detected for each protein. The obtained results (Figure 1) provide evidence that the phosphorylation of STAT3 at Y705 occurs in the analyzed cell lines at different times and seems to be related to the specific characteristic of each cell type. In fact, Y705–STAT3 phosphorylation occurs rapidly in MDA-MB 468, HepG2, and LNCaP cell lines, while in the MCF-7 cell line, it occurs later. STAT3 phosphorylation on Y705 may be the result of several specific activation pathways. The activation of the different receptors taken into account was evaluated in the different cell lines after β-HCH treatment and the results indicate that their phosphorylation occurs within the same time as STAT3 activation, with the exception of MCF-7 cells (Figure 1). The delayed activation of pY705–STAT3 observed in MCF-7 cells is not directly related to the HER2 receptor but seems to be mediated by the JAK2 pathway (Figure S3 of supplementary data). On the contrary, in LNCaP cells, the JAK2, EGFR, and HER2 receptors, although expressed, do not result phosphorylated in response to β-HCH treatment, while SRC is phosphorylated, and thus seems to be directly activated by β-HCH (Figure S4 of supplementary data).

In order verify the action of β-HCH thought cell-specific STAT3-mediated pathways, all cell lines were exposed to 10 μM β-HCH, pretreated or not with specific inhibitors of the different receptors/cytoplasmic tyrosine kinases taken into consideration. In particular, we used Dasatinib as Src inhibitor [28], AZD1480 as JAK2 inhibitor [29], Gefitinib as EGFR inhibitor [30], and Lapatinib as HER2 inhibitor [31].

**Figure 2:**
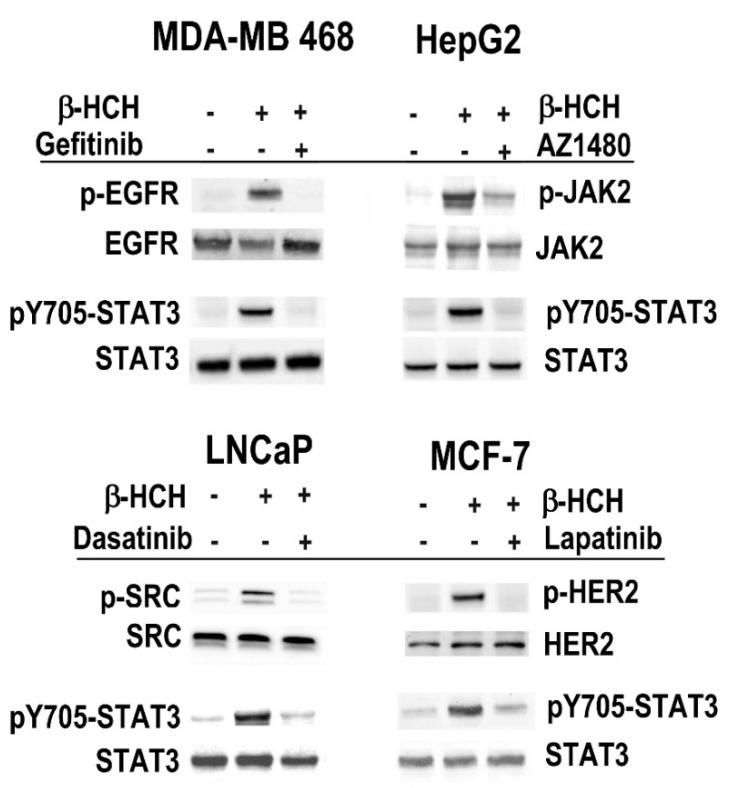
Inhibition of specific signaling pathways activated by β-HCH. Each cell line was treated with 10 μM β-HCH in the presence or absence of specific inhibitors: Gefitinib as EGFR inhibitor in MDA-MB 468 cells; AZD1480 as JAK2 inhibitor in HepG2 cells; Dasatinib as SRC inhibitor in LNCaP cells; and Lapatinib as HER2 inhibitor in MCF-7 cells. Cells were incubated (15 min for MDA-MB 468, HepG2, and LNCaP cells and 2 h for MCF-7 cells) and cellular extracts were subjected to immunoblot analysis. Detection of the unmodified and corresponding phosphorylated form was carried out using specific antibodies.

In this kind of experiment, a single incubation time with β-HCH was selected for each cell line according to the previously obtained results evaluating Y705–STAT3 phosphorylation. In particular, we choose 15 min of β-HCH treatment for MDA-MB 468, HepG2, and LNCaP cells, and 2 h for MCF-7 cells. Total protein extracts were analyzed by Western blotting using specific antibodies for protein receptors and STAT3, both unphosphorylated and phosphorylated forms. Immunoblotting results show the absence of STAT3 and receptors phosphorylation in cells pre-treated with the specific inhibitors (Figure 2). These preliminary results support the hypothesis that STAT3 protein can mediate both a rapid and a delayed cellular response to β-HCH through a non-genomic activity, which involves different cellular pathways, distinctive of each cell line analyzed.

### 2.2. STAT3: Signal Transducer and Transcription Factor Mediating the Activity of β-HCH

To further confirm the role of STAT3 in the cellular response to β-HCH, the expression profile of STAT3 specific target genes, which represents a different phase of the carcinogenesis process (*P21* for cell cycle, *CRP* for inflammation, *BIRC5-Survivin* for apoptosis, and *c-MYC* for proliferation) was evaluated. The same analysis was performed in the presence of the specific STAT3 inhibitor S3I-201 [32].

**Figure 3:**
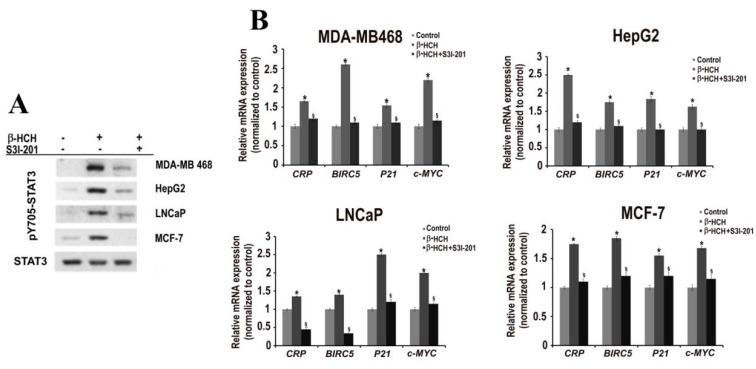
(**A**) Immunoblot analysis of pY705–STAT3 in extracts obtained from MDA-MB 468, HepG2, LNCaP, and MCF-7 cells treated with β-HCH (10 μM) for 4 h in the presence or absence of a specific STAT3 inhibitor (S3I-201). Detection of phosphorylated and unmodified STAT3 was carried out using specific antibodies. (**B**) RT-qPCR analysis of STAT3 target genes (*CRP*, *BIRC5*, *p21*, and *c-MYC*). Analysis was performed on β-HCH treated or untreated cells, as well as on cells pre-incubated with a specific STAT3 inhibitor (S3I-201) before β-HCH treatment. Statistically significant differences (*p* < 0.05) between β-HCH treated and β-HCH untreated cells are marked by *, while statistically significant differences (*p* < 0.05) between β-HCH treated and pre-incubated or not with a specific STAT3 inhibitor are marked by §.

All cell lines were treated with β-HCH in the presence or absence of S3I-201 and the time points of treatment were selected taking into account the rapid or delayed STAT3 activation, previously observed in each cell line. RNAs were purified and analyzed by RT-qPCR and the results are reported in Figure 3A. These results seem to confirm that STAT3 is involved in the cellular response to β-HCH and can probably mediate its potential tumor activity. In fact, in all cell lines tested, β-HCH treatment leads to an increase in the expression level of the STAT3-specific genes analyzed. As expected, this increase was not observed when cells were co-treated with the STAT3 inhibitor. As control, STAT3 activation (as pY705–STAT3) was checked by Western blot in all the cell lines utilized and the pre-treatment with S3I-201 significantly reduced STAT3-phosphorylation (Figure 3B). The overall results support the hypothesis that STAT3 is involved in pathways triggered by β-HCH and that it can mediate the inflammatory, anti-apoptotic, and proliferative activity of this organochlorine compound.

### 2.3. STAT3 as a Master Regulator of the Cell Metabolism

It is well known that POPs are potentially responsible for stressing conditions such as stress-oxidative phenomena [33]. First of all, we decided to check whether β-HCH also could induce oxidative stress, thus, we analyzed reactive oxygen species (ROS) production treating two cell lines, HepG2 and MCF7, characterized by a rapid and delayed cellular response to β-HCH, with 10 μM β-HCH. Tert-butyl hydroperoxide was used as positive control. In both cell lines, the ROS levels were not remarkably increased (Figure S5 in supplementary data), likewise for previous data obtained on dopaminergic neurons treated with 25 μM β-HCH [25]. However, a slight increase was observed in MCF-7 cells. As the above-mentioned approach only investigates the ROS production, we also evaluated the reduced/oxidized glutathione (GSH/GSSG) ratio to assess the β-HCH capability to induce an oxidative stressing condition in the analyzed cell lines. Obtained data demonstrated a different cellular behavior respect to ROS generation (Table S1 in supplementary data). In HepG2 cells, there was already a decrease in the GSH/GSSG ratio after 1 h of treatment, which that could be related to a stressing condition even in absence of detectable ROS generation. Instead, in MCF-7 cells, this ratio increased after 3 h of treatment followed by a decrease after 6 h, confirming the delayed response to β-HCH of this cell line.

Demaria and Poli described the involvement of STAT3 in cellular stress-oxidative responses through the activation of a non-canonical pathway. In this condition, STAT3 results phosphorylated on Ser727 and localized into mitochondria [34,35]. Additionally, STAT3 can interact with specific protein partners, as PKM2 (pyruvate kinase isozyme M2) and HIF-1α (hypoxia-inducible factor-1), both involved in cell energy metabolism [36,37]. PKM2 is an isoform of pyruvate kinase that catalyzes the penultimate step of glycolysis and is preferentially expressed in cancer cells. PKM2 was recently shown to be critical for a metabolic reprogramming with adjustable activity and dynamic cellular re-localization, shuttling from cytoplasm to nucleus. HIF-1α has been largely studied for its role in cell survival in hypoxic conditions and as a player in pathways activated by ROS. As described by DeMaria et al. [15], PKM2 and HIF-1α, together with protein STAT3, seem to establish a vicious “circuit” that is involved in the Warburg effect. This metabolic shift towards aerobic glycolysis (transformation of pyruvate into lactate instead of acetylCoA) is a survival response of tumour cells under unfavorable environmental conditions and represents an index of malignancy and tumour aggressiveness.

**Figure 4:**
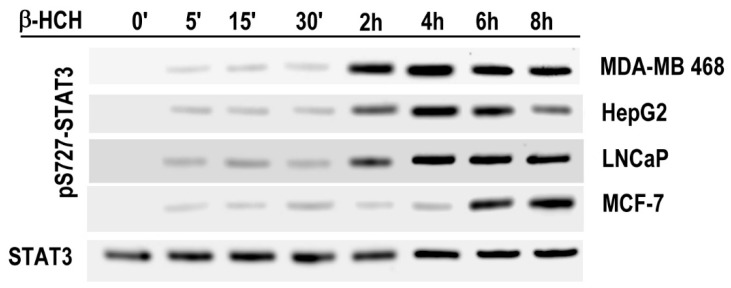
Immunoblot analysis of pS727-STAT3 in MDA-MB 468, HepG2, LNCaP, and MCF-7 cells after incubation with 10 μM β-HCH at different times.

In order to prove whether STAT3 can act as a master regulator of energy metabolism in cellular response to β-HCH, S727–STAT3 phosphorylation was evaluated by Western blotting analysis on total cellular extracts (Figure 4). In each cell line analyzed, pS727–STAT3 phosphorylation is successive to Y705-STAT3 phosphorylation induced by β-HCH treatment, and the delay time seems to be related to the onset of a cell response to β-HCH. In fact, pS727–STAT3 phosphorylation and the GSH/GSSG ratio decrease appear temporally associated.

**Figure 5:**
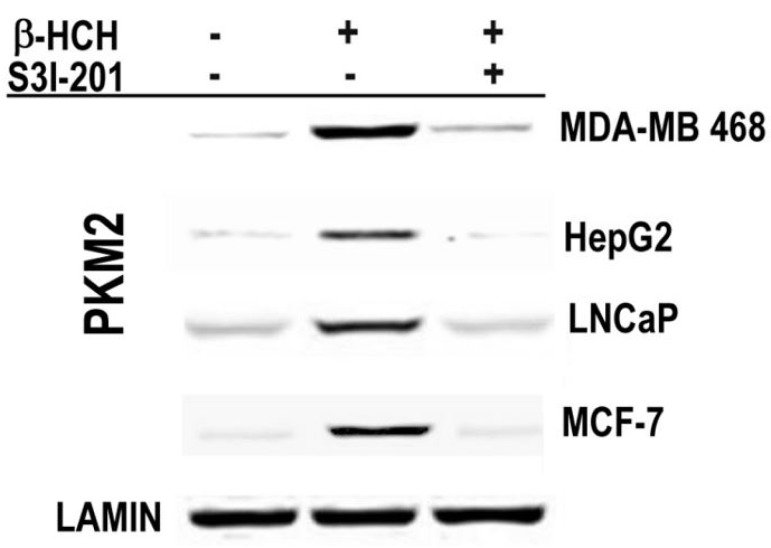
Immunoblot analysis of PKM2 in the nuclear extracts obtained from MDA-MB 468, HepG2, LNCaP, and MCF-7 cell lines treated with β-HCH (10 μM) for 4 h in the presence or absence of a specific STAT3 inhibitor (S3I-201). Lamin was used as specific nuclear marker and normalization protein.

To further confirm whether STAT3 mediates the described vicious cycle, including HIF-1α and PKM2, typical of the Warburg Effect, the nuclear localization of PKM2 was verified, as well as the cellular amount of HIF-1α, which was analyzed after β-HCH treatment either in the absence or presence of STAT3 specific inhibitor S3I-201. The nuclear localization of PKM2, related to the metabolic reprogramming that occurs after oxidative stress induced by β-HCH, was verified by analyzing the nuclear extracts obtained from cultured cells after treatment with β-HCH in presence or absence of S3I-201. Incubation time with β-HCH—4 h for MDA-MB 468, HepG2, and LNCaP cells, and 8 h for MCF-7 cells—was selected taking into account the rapid or delayed STAT3 activation previously observed in each cell line. Figure 5 shows the Western blot analysis of the nuclear extracts obtained from β-HCH treated cells with or without the STAT3 inhibitor. Translocation of PKM2 into the nucleus appears following STAT3 activation in cells treated with β-HCH, but this cannot be longer observed in cells co-treated with S3I-201.

**Figure 6:**
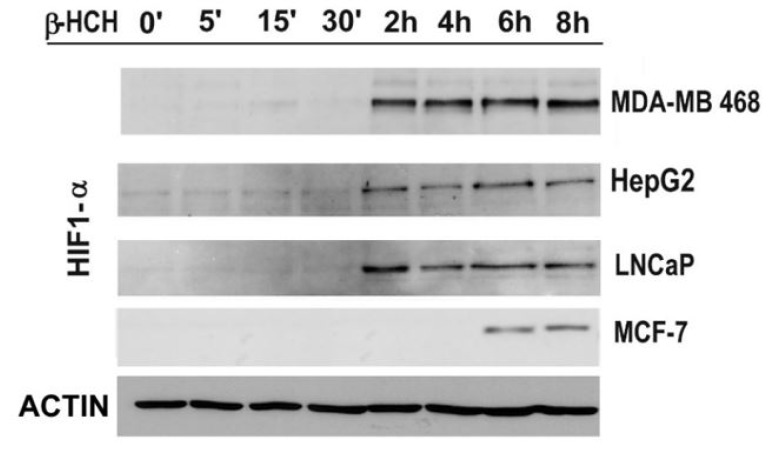
The expression levels of HIF-1α were detected by immunoblot analysis in a time-course experiment performed on MDA-MB 468, HepG2, LNCaP, and MCF-7 cells treated with β-HCH (10 μM). Actin was used as normalization protein.

The expression level of HIF-1α was evaluated in the considered cell lines, after treatment with β-HCH, through a time-course analysis from 5 min to 8 h. The immunoblot shows that the cellular amount of HIF-1α follows the same trend of pS727–STAT3 modification (Figure 6). In fact, HIF-1α cannot be detected by Western blot in control cells and became visible after 2 h of β-HCH-treatment in LNCaP, MDA-MB 468, and HepG2 cells, and after 6 h of treatment in MCF-7 cells.

**Figure 7:**
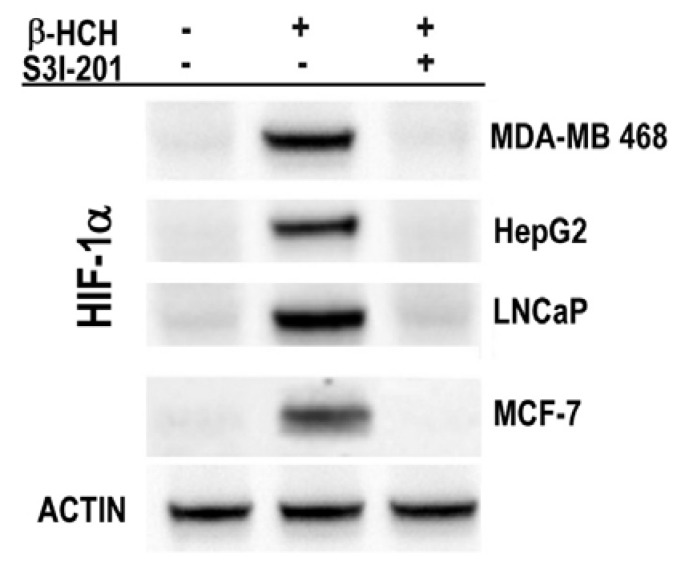
Immunoblot analysis of HIF-1α expression in MDA-MB 468, HepG2, LNCaP, and MCF-7 cells after 8 h treatment with 10 μM β-HCH. Analysis was performed after 8 h treatment with 10 μM β-HCH, as well as on cells pre-incubated with a specific STAT3 inhibitor (S3I-201) before β-HCH treatment. Actin was used as normalization protein.

The correlation between STAT3 and HIF-1α was highlighted using the STAT3 inhibitor S3I-201. In this experiment, the β-HCH incubation time was the same, selected to verify the nuclear localization of PKM2. Immunoblot clearly shows the relative absence of HIF-1α in cells treated with both β-HCH and STAT3 inhibitor S3I-201 (Figure 7).

The above reported data allow us to hypothesize that STAT3 could activate the “feedback loop” with PKM2 and HIF-1α described by De Maria et al. [15], also in response to β-HCH, thus influencing energy metabolism.

**Figure 8:**
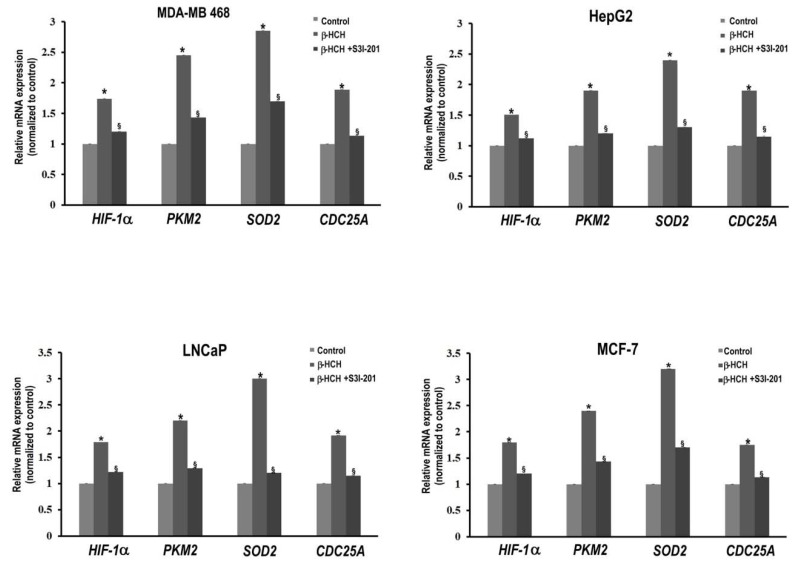
RT-qPCR analysis of STAT3 target genes involved in oxidative-stress and energy metabolism (*PKM2*, *HIF-1α*, *SOD2*, and *CDC25A*). Analysis was performed after 8 h treatment with 10 μM β-HCH, as well as on cells pre-incubated with a specific STAT3 inhibitor (S3I-201) before β-HCH treatment. Statistically significant differences (*p* < 0.05) between β-HCH treated and β-HCH untreated cells are marked by *, while statistically significant differences (*p* < 0.05) between β-HCH treated and pre-incubated or not with a specific STAT3 inhibitor are marked by §.

The expression profiles of both HIF-1α and PKM2 genes were tested in response to β-HCH treatment, as well as two other STAT3 target genes [38,39], such as SOD2 (mitochondrial superoxide dismutase 2)—an indicator of oxidative stress—and CDC25A (cell division cycle 25 homolog A), to further confirm the existence of a STAT3–PKM2 axis in the Warburg Effect. SOD2 is an antioxidant enzyme that plays an important role in defense against reactive oxygen species and is overexpressed under stress conditions. CDC25A is a protein phosphatase key regulator of the cell-cycle that is overproduced in many human cancers. In particular, it has recently been reported that the phosphatase activity of CDC25A contributes to PKM2 nuclear translocation [40]. Because CDC25A is under STAT3 regulation, its activity could be involved in the regulation of the “feedback loop” mentioned above. RT-qPCR experiments to evaluate the gene expression profiles confirmed the upregulation of the four genes analyzed in cells treated with β-HCH, but this cannot be observed in the same cells treated in the presence of the STAT3 inhibitor (Figure 8)

## 3. Discussion

Environmental pollution is one of the most serious problems faced by mankind today. In the last few years, great attention has been paid in evaluating the adverse impact of endocrine disrupting chemicals (EDCs) on human health, and the cytotoxic mechanisms of organochlorine compounds, such as dioxin and polychlorinated biphenyls, have been widely described in literature [41].

The β-isomer of hexaclorocyclohexane has not been largely investigated, despite its relatively common geographic distribution [3] and its physicochemical properties (great stability, environmental persistence, high lipid solubility, bioaccumulation) make it potentially dangerous. A detailed environmental–epidemiological study carried out on exposed population living throughout the “Valle del Sacco”—south of Rome [23,24], as well as others reported by different countries [42], indicates correlations between the incidence of a wide range of diseases and the occurrence of β-HCH contamination.

Therefore, this research aimed to clarify some of the molecular mechanisms at the basis of the alteration of key signaling pathways triggered by high levels of β-HCH, such as those revealed in human blood of exposed people.

The obtained results support the hypothesis that STAT3 is involved, as a hub protein, in several cellular pathways affected by β-HCH. STAT3 phosphorylation on residue Y705 was observed in all evaluated cell lines in response to β-HCH stimulation and occurs within 15 min in MDA-MB 468, HepG2, and LNCaP cells, and with a 2 h delay in MCF-7 cells. The activated pY705–STAT3 can translocate into the nucleus and execute its transcriptional functions upregulating target genes, as shown by RT-qPCR analysis. In addition, our research provided evidence that STAT3 may also acts as a master regulator of energy metabolism under the oxidative stress conditions induced by β-HCH. As reported in previous studies, this cellular condition seems to be one of the major determinants for the metabolic reprogramming typical of the Warburg Effect [43,44].

In cell lines that exhibit a rapid response to β-HCH through the activation of STAT3 canonical pathway (pY705-STAT3), the phosphorylation of STAT3 on Serine 727 occurs after 2 h of treatment; otherwise, in MCF-7 cells, where there is a delayed activation of STAT3, S727–STAT3 phosphorylation results after 6 h. The observation that S727–STAT3 phosphorylation always occurs about 2 h later than Y705–STAT3 phosphorylation lead us to hypothesize that the canonical pathway of STAT3 (pY705) may activate a non-canonical pathway, characterized by pS727 modification, that can be involved in cell metabolism regulation and Warburg effect. In fact, pY705 modification triggers STAT3 as signal transduction and transcription factor leading to an increased expression of genes involved in cell cycle, inflammation, apoptosis, and proliferation, all related to the carcinogenesis process. All these activated functions require an increased level of energy, and thus a burst in oxidative respiration, which will result in overproduction of ROS. The increased level of SOD2 expression observed after prolonged exposure to β-HCH may support the hypothesis that β-HCH can induce, after an early STAT3 activation (pY705–STAT3), an oxidative stress condition. Under our experimental conditions, β-HCH treatment does not produce a remarkable increase in ROS levels. However, a slight increase in ROS induced by β-HCH can be observed. Moreover, the GSH/GSSG ratio decreases following β-HCH treatment, which may be related to the induction of cellular stressing condition or the activation of intracellular pathways altering the cellular redox state. Literature evidence indicates that STAT3 is a redox sensitive protein, but the effect of oxidative stress on STAT3 regulation is still confusing. ROS may directly trigger protein phosphorylation and thus up-regulate STAT3 activity, as well as may induce oxidation of STAT3, hindering its transcriptional activity [45]. However, we showed here that β-HCH treatment can trigger Y705–STAT3 phosphorylation through the activation of different canonical cellular pathways in the analyzed cell lines. We also show that S727–STAT3 phosphorylation is a consequence of pY705–STAT3 activation and is probably related to HIF-1α up-regulation through a non-canonical pathway. We cannot exclude that β-HCH can induce an oxidative stressing condition straight after activating STAT3, but our data suggest that both canonical and non-canonical pathways involving STAT3 are activated by β-HCH treatment.

S727–STAT3 phosphorylation is a hallmark of stressing conditions and may lead the cell to modify its energy metabolism, avoiding the production of an elevated level of harmful ROS and supplying itself with an adequate energy production [34,35]. Additionally, the overexpression of HIF-1α and PKM2 has been found when STAT3 is phosphorylated on S727, supporting the evidence of a feedback loop between STAT3, HIF-1α, and PKM2 in response to ROS accumulation.

A further confirmation of the role of STAT3 as a hub in cellular responses to β-HCH is also confirmed by the overexpression of CDC25A observed after β-HCH treatment. As above mentioned, CDC25A phosphatase activity can regulate the nuclear translocation of PKM2. In fact, the cytosolic form of PKM2 is tetrameric and phosphorylated. Upon dephosphorylated, PKM2 can dissociate in dimers and then translocate into the nucleus, where it can phosphorylate STAT3, maintaining it in an activated form. STAT3 may act as transcription factors for HIF-1α, which in turn may activate the expression of PKM2, triggering the described “vicious circle” sustained by HIF-1α, STAT3, and PKM2 [15]. Considering that CDC25A gene is also under STAT3 control [40], we found it interesting to verify its expression profile under β-HCH treatment. CDC25A is upregulated in response to β-HCH, which seems to be STAT3-dependent, because the co-treatment with the STAT3 inhibitor S3I-201 abolishes this effect. Taking into account all these considerations, it is conceivable to hypothesize that through CDC25A, STAT3 establishes a positive feedback with PKM2 that enhances cellular responses to β-HCH. This observation is a further confirmation of the crucial role played by STAT3, both as crossroads of the different signaling pathways and as a master regulator of cell energy metabolism.

## 4. Materials and Methods

### 4.1. Cell Cultures

Human prostate cancer cell line LNCaP, human breast cancer cell lines MCF-7 and MDA-MB 468, and human hepatoma cell line HepG2 were obtained from American Type Culture Collection (ATCC). Cells were grown to 80% confluence at 37 °C in 5% CO_2_ in the appropriate culture medium, RPMI 1640 (Sigma-Aldrich, Milano, Italy) or DMEM-LG (Sigma-Aldrich), supplemented with 1% sodium pyruvate, 10% fetal bovine serum, 2 mM glutamine, 100 μg/mL streptomycin, and 100 U/mL penicillin.

Beta-hexaclorocyclohexane (β-HCH) (Sigma-Aldrich, 33376), at a final concentration of 10 μM, was tested on each cell line pre-treated or not with specific inhibitors: 6 μM AZD1480 (Sigma-Aldrich, SML1505), 100 μM S3I-201 (Sigma-Aldrich, SML0330), 70 nM Dasatinib (Selleckchem, Roma, Italy, Cat. No. S1021), 0.8 μM Lapatinib (Sigma-Aldrich, CDS022971), and 15 μM Gefitinib (Sigma-Aldrich, SLM1657).

The β-HCH cytotoxicity was evaluated by seeding cells in 96-well plates and measuring cell viability after 24 and 48 h incubation in the presence of different concentrations of β-HCH (5, 10, 25, 50, 75, 100, 125, 150, 175, and 200 μM). Cell viability was measured using MTT (3-(4,5-dimethylthiazol-2-yl)-2,5-diphenyl-2H-tetrazolium bromide) (Sigma-Aldrich, M2128). Briefly, the culture medium was removed and 125 μL of MTT solution (0.5 mg/mL MTT in culture medium) was added to each well. After 3 h incubation, the solution was removed and the insoluble formazan dye resulting from the conversion of tetrazolium salt by metabolically active cells was dissolved by adding 125 μL/well of DMSO and measured at 570 nm using the Appliskan plate reader (Thermo Scientific, Monza, Italy).

To assess the effect of β-HCH on cell proliferation, cells were seeded on 6-well plates and treated with 10 μM β-HCH. Cell number was evaluated after 24 and 48 h incubation.

Reactive oxygen species generated by stressing cells with β-HCH were quantified using the CellROX™ Green Flow Cytometry Assay Kit (Thermo Fisher Scientific, Rodano, Italy, C10492) following manufacturer’s instructions. Tert-butyl hydroperoxide was used as positive control. Samples were analyzed by a BD Accuri C6 flow cytometer (BD Biosciences).

Reduced (GSH) and oxidized (GSSG) glutathione were measured by HPLC-UV. Briefly, cell pellets (1 × 10^6^) were suspended in 10% ice-cold TCA and centrifuged for 15 min at 9000× *g*. The supernatant was collected and GSH and GSSG were measured by HPLC with UV detection at 215 nm. The separation was achieved using a poroshell 120 EC-C18 column (3 × 150 mm, 2.7 μm) at a flow rate of 0.8 mL/min with the following elution gradient: 0–3 min 100% A + 0% B, 3–10 min from 100% A to 100% B. The composition of mobile phase A was 0.1% trifluoroacetic acid in water and mobile phase B was 0.1% trifluoroacetic acid in water/acetonitrile (93:7). In these chromatographic conditions, retention times were 2.58 min and 7.01 min for GSH and GSSG, respectively.

### 4.2. Proteins Extraction and Immunoblotting

Cells cultured on 6-well plates were scraped, harvested by centrifugation, and washed in PBS. Total protein extracts were obtained using a lysis buffer containing 2% SDS, 20 mM Tris-hydrocloride Ph = 7.4, 2 M urea, 10% glycerol added with 2 mM sodium orthovanadate, 10 mM DTT, and a protease inhibitors cocktail diluted 1:100 (Sigma-Aldrich). Nuclei were obtained from cell pellets using a hypotonic buffer (10 mM HEPES, 10 mM KCl, 1.5 mM MgCl2, 0.5 mM DTT) added with 0.05% Triton-X, 2 mM sodium orthovanadate, and a protease inhibitors cocktail diluted 1:100 (Sigma-Aldrich). Thus, nuclei were harvested by centrifugation and washed in hypotonic buffer, and nuclear protein extracts were obtained as described above for total protein extracts. Proteins were resolved by SDS-PAGE 10% TGX FastCast™ Acrylamide gel (BioRad, Segrate, Italy) and transferred on PVDF membranes (BioRad) using Trans-Blot^®^ Turbo™ Transfer System (BioRad). The membranes were blocked with 3% *w*/*v* non-fat dried milk or 0.2% *w*/*v* I-block (Thermo Fisher Scientific, Rodano, Italy) in Tris-buffered saline containing 0.05% Tween-20 (TBS-T) and incubated with a specific primary antibody for 1 h. Subsequently, membranes were washed three times in TBS-T, and then incubated for an additional hour with appropriate horseradish peroxidase- or alkaline phosphatase-conjugated secondary antibody (Jackson ImmunoResearch, Pero, Italy). The peroxidase signal was detected with ECL Fast Femto reagent (Immunological Science, Roma, Italy), acquired by Molecular Imager^®^ ChemiDoc™ MP System (Bio-Rad), and the intensity of protein bands was quantified using the ImageLab Software. The alkaline phosphatase signal was detected with BCIP/NBT reagents (Carl Roth, Milano, Italy, CAS No. 298-83-9 and 6578-06-9). β-actin (total extracts) or lamin (nuclear extracts) were used as normalization protein.

The immunoblotting detection was carried out using anti-PKM2 (Cell Signaling D78A4, antibody dilution 1:1000), anti- HIF-1α (Invitrogen, Monza, Italy, MA1-516, antibody dilution 1:2000), anti-pY705STAT3 (Cell Signaling D3A7, antibody dilution 1:2000), anti-pS727STAT3 (Cell Signaling, Danvers, MA, USA 9134S, antibody dilution 1:1000), anti-JAK2 (Cell Signaling D2E12, antibody dilution 1:1000), anti-PY1007/1008JAK2 (Cell Signaling C80C3, antibody dilution 1:1000), anti-EGFR (Cell Signaling D38B1, antibody dilution 1:1000), anti-pY1173EGFR (Cell Signaling 53A5, antibody dilution 1:1000), anti-Src (Cell Signaling 32G6, antibody dilution 1:1000), and anti-pY416Src (Cell Signaling 6943S, antibody dilution 1:1000) primary antibodies. Each experiment was replicated at least three times.

### 4.3. Extraction of RNA and RT-qPCR

Total RNA was extracted from cells using TRIzol reagents (Immunological Science) in accordance with the manufacturer’s instructions. RNA was quantified spectrophotometrically and its quality was assessed by 1% agarose gel electrophoresis and staining with ethidium bromide. The reverse transcription was carried out with Super Script II R-Transcriptase (FS-RT-3022, Fisher Molecular Biology, Rodano, Italy). Gene expression was evaluated with specific primers for CDC25A, BIRC-5, c-MYC, CRP, p21, HIF-1α, PKM2, and S18 (housekeeping) (all from Qiagen S.r.l., Milano, Italy) using CFX Connect™ Real-Time PCR Detection System (BioRad) with a SYBR green fluorophore based real-time reaction (Brilliant SYBR Green QPCR Master Mix, Thermo Fisher Scientific). Expression data were analyzed using CFX Manager™ Real Time PCR Detection System Software, Version 3.1 (BioRad).

### 4.4. Statistical Analysis

The repeatability of results was confirmed by performing all experiments at least three times. The obtained values are presented as mean and standard deviation. Statistical analysis was performed with GraphPad Prisma software using Student’s *t*-test.

## 5. Conclusions

**Figure 9:**
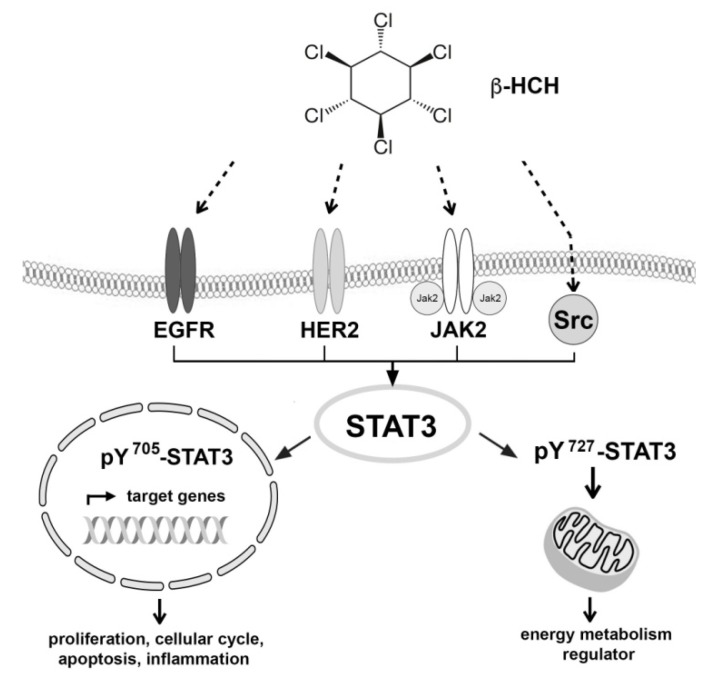
The central role of STAT3 as a hub protein in signaling pathways triggered by β-HCH.

In conclusion, our results suggest the STAT3 involvement in β-HCH toxicity both through canonical and non-canonical pathways. Thus, STAT3 may regulate the cell response to β-HCH switching from an acute to chronic phase. This process may be responsible for the transformation of the tumor in a more aggressive stage, and this can be testified by the metabolic shift towards the aerobic glycolysis (Figure 9).

The obtained results support the evidence that STAT3 is involved, as a hub protein, in the acute and chronic toxicity induced by β-HCH. On the basis of these results, it could be conceivable to hypothesize STAT3 as a new valid cellular target to develop therapeutic strategies based on the inhibition of STAT3-mediated pathways, in addition to conventional chemotherapy, to deal with tumours in patients exposed to POPs (i.e., β-HCH).

Considering the wide number of natural compounds able to modulate STAT3 activity (i.e., silibinin, curcumin, etc.) [46], their protective use can be beneficial for people exposed to such pollutants.

## Supplementary Materials

Supplementary materials can be found at http://www.mdpi.com/1422-0067/19/7/2108/s1.

## Abbreviations

OCPs	Organochlorine pesticides
β-HCH	β-hexaclorocyclohexane
POPs	Persistent Organic Pollutants
EDCs	endocrine disrupting chemicals
