# Supplementary material for: STAT3, a Hub Protein of Cellular Signaling Pathways, Is Triggered by β-Hexaclorocyclohexane
Source: Int J Mol Sci. 2018 Jul 20;19(7):2108. doi: 10.3390/ijms19072108 (PMC6073614; doi:10.3390/ijms19072108)
Supplement: Supplementary file 1 [file ijms-19-02108-s001.pdf]

International Journal of *Molecular Sciences*

MDPI

Article

STAT3, a Hub Protein of Cellular Signaling Pathways, Is Triggered by β-Hexaclorocyclohexane

Elisabetta Rubini[1], Fabio Altieri[1,2], Silvia Chichiarelli[1], Flavia Giamogante[1], Stefania Carissimi[1], Giuliano Paglia[1], Alberto Macone[1] and Margherita Eufemi[1,2,*].

[1] Department of Biochemical Sciences "A. Rossi Fanelli", Sapienza University ", P.le A. Moro 5, 00185, Rome, Italy

[2] Istituto Pasteur-Fondazione Cenci Bolognetti, Sapienza University ", P.le A. Moro 5, 00185, Rome, Italy

* Correspondence: margherita.eufemi@uniroma1.it; Tel.: +39-06-49910598

Appendix A

Supplementary data.

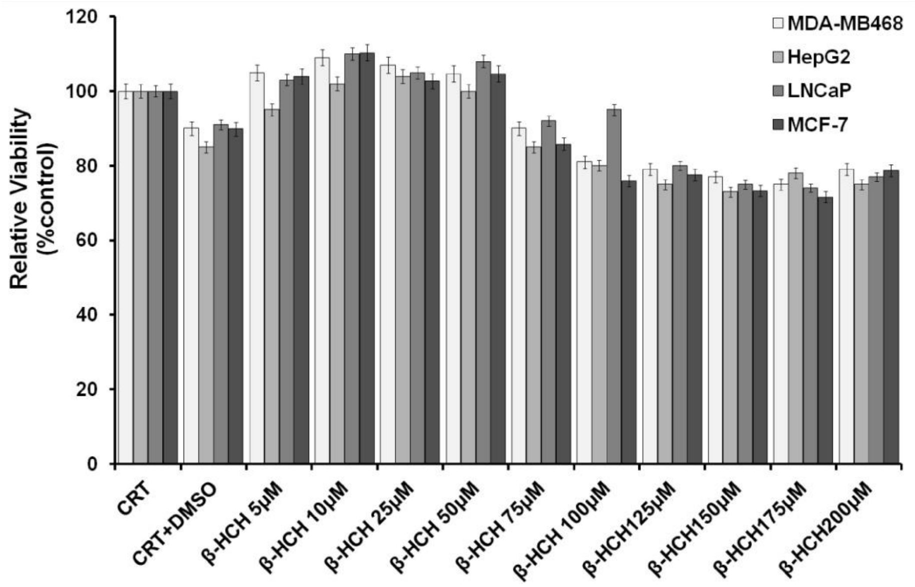

Figure S1. Cell viability assay performed on human prostate cancer (LNCaP), human breast cancer (MCF-7 and MDA-MB 468), and human hepatoma (HepG2) cell lines after 48h treatment with increasing concentration of β-hexaclorocyclohexane (β-HCH). 0.3% DMSO has also been tested as control.

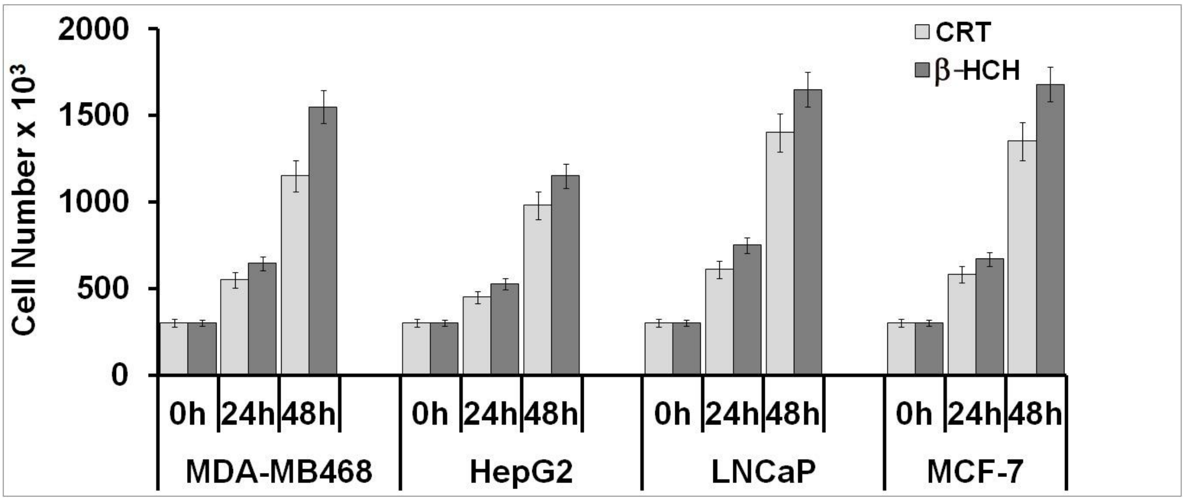

Figure S2 Evaluation of MDA-MB 468, HepG2, MCF-7, and LNCaP cell proliferation at 24h and 48h of treatment with 10 μM β-HCH.

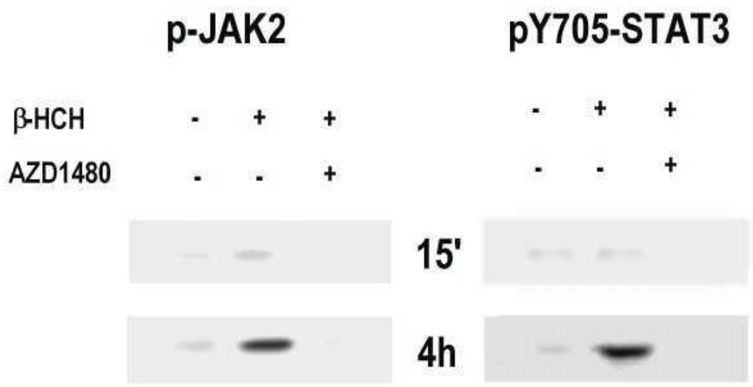

Figure S3. Evaluation of JAK2 and signal transducer and activator of transcription 3 (STAT3) phophorylation level in MCF-7 cell line treated with 10 μM β-HCH. Cellular extracts were obtained from MCF-7 cells incubated for 15 minutes and 4 hours in the absence or presence of JAK2 inhibitor (AZD1480), and then subjected to immnunoblot analysis.

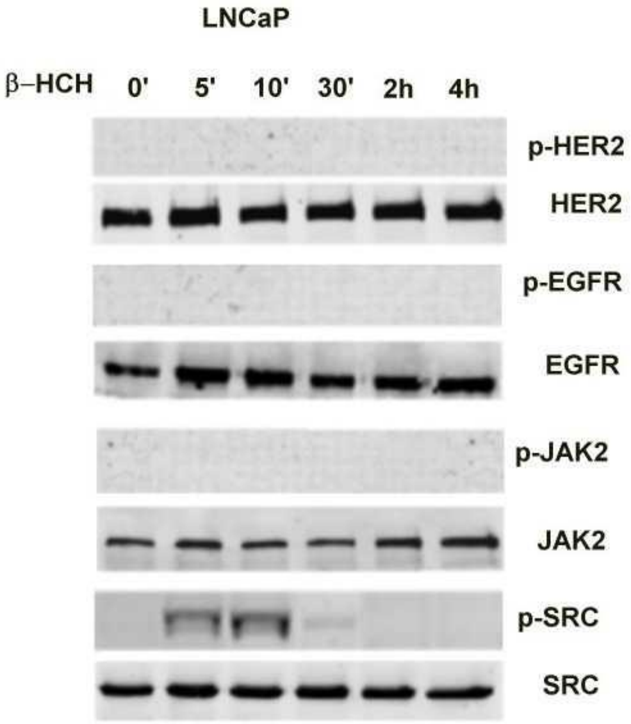

Figure S4. Analysis of the signaling pathways triggered by β-HCH in LNCaP cells. Immunoblot analysis of HER2, EGFR, JAK2, and SRC protein in a time-course assay. Both the unmodified and phosphorylated forms of each protein were evaluated.

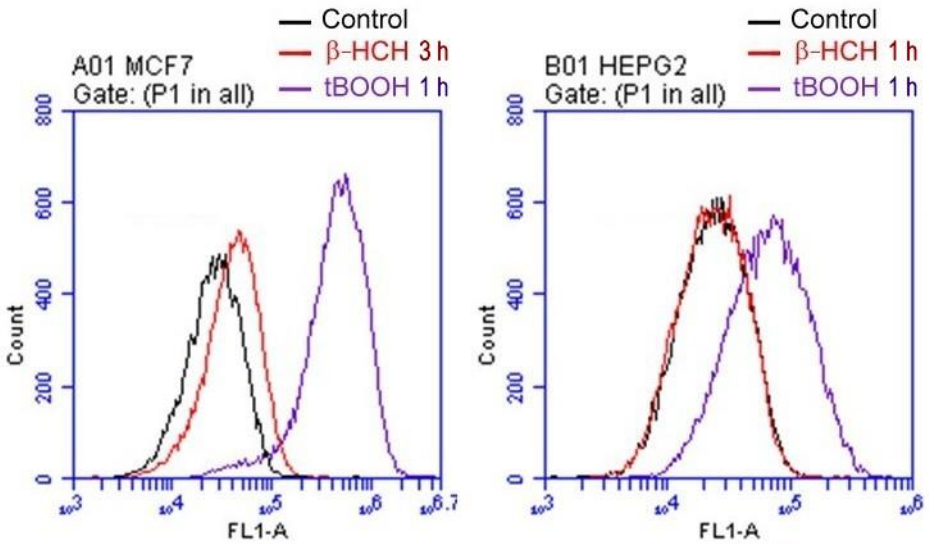

Figure S5. Quantification of ROS generated in MCF-7 (left) and HepG2 (right) cells treated with 10 μM β-HCH and 75 μM tert-butyl hydroperoxyde.

Table S1. Quantification of reduced glutathione (GSH), oxidized glutathione (GSSG), and ROS in human breast cancer (MCF-7) and human hepatoma (HepG2) cells treated for different times with 10 μM β-hexaclorocyclohexane (β-HCH).

	**GSH** (nmol)	**GSSG** (nmol)	**GSH/GSSG** ratio	**Cell ROS** (mean fluorescence)
**HepG2 Control**	30.16	4.31	7.00	28313.32
**HepG2 β-HCH** 10 **μM 1h**	30.28	6.20	4.88	27622.26
**HepG2 β-HCH** 10 **μM 3h**	30.65	6.47	4.74	26095.56
**MCF-7 Control**	40.03	4.71	8.49	30535.25
**MCF-7 β-HCH** 10 **μM 3h**	48.43	4.02	12.06	46279.62
**MCF-7 β-HCH** 10 **μM 6h**	46.58	7.55	6.17	39146.35

© 2018 by the authors. Submitted for possible open access publication under the terms and conditions of the Creative Commons Attribution (CC BY) license (http://creativecommons.org/licenses/by/4.0/).
